# Red Blood Cell‐Mediated Enhancement of Hematopoietic Stem Cell Functions via a Hes1‐Dependent Pathway

**DOI:** 10.1096/fj.202500885R

**Published:** 2025-09-09

**Authors:** Erika Yamashita, Soichiro Hashimoto, Hiroaki Abe, Takao Sudo, Daisuke Okuzaki, Toshiya Okawa, Masaru Ishii

**Affiliations:** ^1^ Department of Immunology and Cell Biology, Graduate School of Medicine and Frontier Biosciences The University of Osaka Osaka Japan; ^2^ WPI–Immunology Frontier Research Center The University of Osaka Osaka Japan; ^3^ Life–Omics Research Division, Institute for Open and Transdisciplinary Research Initiative The University of Osaka Osaka Japan; ^4^ Laboratory of Bioimaging and Drug Discovery, National Institutes of Biomedical Innovation, Health and Nutrition Osaka Japan; ^5^ StemRIM Institute of Regeneration–Inducing Medicine The University of Osaka Osaka Japan; ^6^ Department of Hematology and Oncology, Graduate School of Medicine The University of Osaka Osaka Japan; ^7^ Department of Haematology, National Hospital Organization Osaka National Hospital Osaka Japan; ^8^ Genome Information Research Center, Research Institute for Microbial Diseases The University of Osaka Osaka Japan

**Keywords:** bone marrow, erythrocyte, hematopoiesis, hematopoietic stem cell, Hes1, red blood cell

## Abstract

In bone marrow, cell numbers are balanced between production and loss. After chemotherapy, blood cell counts decrease initially but later recover as hematopoietic progenitor cells expand, although the mechanisms underlying this recovery are still unclear. We investigated the influence of red blood cells (RBCs) on hematopoietic stem cell (HSC) function during bone marrow recovery. Following chemotherapy, RBC concentrations in bone marrow peaked on day 5 posttreatment, coinciding with the recovery of hematopoiesis. Coculture of HSCs with RBCs resulted in a significant increase in hematopoiesis. Direct contact between RBCs and HSCs was essential for enhancement of hematopoiesis, and HSCs precultured with RBCs resulted in greater numbers of donor‐derived mature hematopoietic cells after transplantation. RNA‐sequencing analysis showed that Hes1 was the most significantly upregulated transcription factor in RBC coculture, and the response to RBC‐induced hematopoiesis of Hes1‐deficient HSCs was reduced. These findings imply a role of RBCs and Hes1 in the enhancement of hematopoietic recovery following bone marrow stress.

## Graphical abstract

After chemotherapy, blood cell numbers initially decrease but later recover as hematopoietic progenitor cells increase. Following chemotherapy, RBC concentrations in bone marrow peaked at day 5, and direct contact between HSCs and RBCs increased. This contact induced elevated expression of *Notch1* and *Hes1* in HSCs. *Hes1*‐deficient HSCs and treatment with a Notch1 inhibitor reduced the hematopoietic response to RBCs. These findings imply that RBCs enhance hematopoietic recovery after bone marrow stress through activation of the Notch1–Hes1 signaling axis.

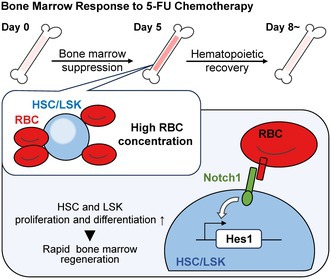

**Abbreviations**
5‐FU: 5‐fluorouracil
Ab: antibody
BFU‐E: burst‐forming unit‐erythroid
BM: bone marrow
cKO: conditional knockout
CLP: common lymphoid progenitor
CMP: common myeloid progenitor
Ctrl: control
DARC: duffy antigen receptor for chemokines
EPO: erythropoietin
EV: extracellular vesicle
GM‐CSF: granulocyte‐monocyte colony‐stimulating factor
GMP: granulocyte/monocyte progenitor
HSC: hematopoietic stem cell
ILC: type 2 innate lymphoid cell
Lin^−^
: lineage negative
LSK: Lin^−^Sca‐1^+^c‐Kit^+^
MEP: megakaryocyte/erythrocyte progenitor
MPP: multipotent progenitor
PCS: Principal component analysis
q‐PCR: quantitative real‐time PCR
RBC: red blood cell
RNA‐seq: RNA‐sequencing
SCF: stem cell factor
SDF‐1: stromal cell‐derived factor 1
SN: supernatant
ST‐HSC: short‐term hematopoietic stem cell
WBCs: white blood cells

## 1 Introduction

Hematopoietic stem cells (HSCs) reside primarily in the bone marrow and umbilical cord blood and supply all blood immune cells throughout life [1, 2, 3, 4, 5]. Bone marrow transplantation is an effective therapeutic approach for intractable hematopoietic disorders such as leukemia [6, 7]; however, insufficient engraftment caused by the limited number of donor cells is a serious problem, and securing a stable donor source is critical. Although the factors that characterize HSCs and their microenvironmental niche have been investigated [8, 9, 10], no ex vivo bone marrow HSC amplification system has yet received approval for clinical application [11, 12].

Bone marrow cell numbers are usually maintained at a constant level, with a balance between the cells produced and those that are lost. When a patient undergoes chemotherapy, the number of blood cells in the bone marrow initially decreases significantly, but later recovers as the surviving hematopoietic progenitor cells in the bone marrow expand rapidly [13]; however, the mechanism underlying this stress‐induced emergent hematopoiesis remains unclear. We reported previously that type 2 innate lymphoid cells (ILC2s) in bone marrow can sense bone marrow damage after chemotherapy and promote recovery of hematopoiesis by secreting granulocyte–monocyte colony‐stimulating factor (GM‐CSF) [14]. Nevertheless, the number of ILC2s is generally low and many other cell types are affected by bone marrow stress. Therefore, the cellular mechanisms underlying the stress‐induced recovery of hematopoiesis are unclear.

Red blood cell (RBC) numbers in bone marrow are significantly elevated immediately before recovery of hematopoiesis, implying that RBCs enhance the ability of hematopoietic progenitor cells to produce blood cells. In this study, we evaluated the role of RBCs in the recovery of hematopoiesis after bone marrow stress.

## 2 Materials and Methods

### 2.1 Mice

C57BL/6 mice were obtained from CLEA Japan (Tokyo, Japan). The congenic C57BL/6 strain (C57BL/6SJL; CD45.1 alloantigen) was purchased from The Jackson Laboratory (Bar Harbor, ME, USA) and used for transplantation experiments. *Hes1* floxed (B6.Cg‐Hes1^tm1Imayo^/Rbrc) mice purchased from RIKEN BioResource Research Center (Kyoto, Japan) were bred with Vav1–iCre (B6.Cg‐Commd10^Tg(Vav1‐icre)A2Kio^/J) mice purchased from The Jackson Laboratory. Mice were bred and maintained under specific‐pathogen‐free conditions at the animal facilities of Osaka University, and all animal experiments were performed in accordance with the Experimental Animal Guidelines of Osaka University, using approved protocols. This study used 8 to 20‐week‐old female or male mice (no sex‐related differences were confirmed). The mice were randomly housed in groups and selected for the experiments. 5‐Fluorouracil (5‐FU) was purchased from Kyowa‐Hakko Kirin (Tokyo, Japan) and administered intravenously at 150 mg/kg to C57BL/6 mice.

### 2.2 Isolation of Hematopoietic Stem Cells

Total bone marrow cells were collected from the femur and tibia, and mature hematopoietic cells were removed using a Lineage Cell Depletion Kit (Miltenyi Biotec, Bergisch Gladbach, Germany). Cells were stained with Lineage Cell Detection Cocktail‐Biotin (Miltenyi Biotec), PerCP/Cy5.5‐Streptavidin (eBioscience, San Diego, CA, USA, Cat# 45–4317‐82, RRID: AB_10311495), APC‐c‐Kit Ab (BD Biosciences, Franklin Lakes, NJ, USA, Cat# 561074, RRID: AB_10563203), PE/Cy7‐Sca‐1 Ab (BioLegend, San Diego, CA, USA, Cat# 108113, RRID: AB_493597), BV421‐CD48 Ab (BioLegend, Cat# 103427, RRID: AB_10895922) and PE‐CD150 Ab (BioLegend, Cat# 115903, RRID: AB_313682). Lineage‐negative (Lin^−^) Sca‐1^+^ c‐Kit^+^ CD48^−^ CD150^+^ cells were sorted as HSCs using an SH800 cell sorter (Sony, Tokyo, Japan).

### 2.3 Collection of Red Blood Cells and Extracellular Vesicles

Peripheral blood was collected from the heart of a C57BL6/J mouse. Leukocytes were removed by passing the blood through a Plasmadipur filter (Euro‐Diagnostica, Skane Lan, Sweden) and irradiated at a dose of 20 Gy using a gamma ray irradiator (Gamma Cell 40; Atomic Energy of Canada, Chalk River, ON, Canada). After washing with phosphate‐buffered saline (PBS), the precipitated fraction was collected as RBCs. To isolate extracellular vesicles (EVs), the RBCs were immersed in Terumo Blood Bag MAP Solution (Terumo, Tokyo, Japan) at 1.0 × 10^9^ cells/mL and left to stand at 4°C for 2 days. RBCs were harvested by centrifugation, and the supernatant was ultracentrifuged at 44,000 rpm (13 000 × ***g***) for 70 min at 4°C. The precipitated fraction was collected as the RBC‐derived EV fraction (EV) and the supernatant (SN) was collected separately.

### 2.4 Cell Culture

HSCs were cultured with or without RBCs, RBC‐derived EV fraction (EV), supernatant (SN) or microbeads 5 μm in diameter (Supelco, Bellefonte, PA, USA) in Stem Span SFEM Hematopoietic Cell Culture Medium (Veritas Technologies, Santa Clara, CA, USA) with 100 ng/mL mTPO (Peprotech, Cranbury, NJ, USA) and 10 ng/mL mSCF (Peprotech) using 96‐well U‐bottomed plates at 37°C in 5% CO_2_. For noncontact coculture, HSCs were cultured in 24‐well plates and a Transwell system (Corning, Corning, NY, USA). HSC and RBC numbers are reported in the figures.

### 2.5 Flow Cytometry

Cultured cell suspensions were hemolyzed with ACK lysis buffer (Thermo Fisher Scientific, Waltham, MA, USA). Lin^−^ cells, LSK (Lin^−^ Sca‐1^+^ c‐Kit^+^) cells, and CD48^−^ CD150^+^ LSK (Lin^−^ Sca‐1^+^ c‐Kit^+^ CD48^−^ CD150^+^) cells were treated with Fc Blocker (BD Biosciences, Cat# 553141, RRID:AB_394656) and stained with biotin antibody (Ab) cocktail (biotin‐CD45R/B220; BioLegend, Cat# 103203, RRID: AB_312988, biotin‐Gr‐1; BioLegend, Cat# 108403, RRID: AB_313368, biotin‐CD11c; BioLegend, Cat# 117303, RRID: AB_313772, biotin‐Ter119; BioLegend, Cat# 116203, RRID: AB_313704, and biotin‐CD3e; BioLegend, Cat# 100303, RRID: AB_312668), PerCP/Cy5.5‐streptavidin, APC‐c‐Kit Ab, PE/Cy7‐Sca‐1 Ab, BV421‐CD48 Ab, and PE‐CD150 Ab. Common myeloid progenitor (CMP; Lin^−^ c–Kit^+^ Sca‐1^−^ CD16/32^Lo^ CD34^+^), megakaryocyte/erythrocyte progenitor (MEP; Lin^−^ c–Kit^+^ Sca‐1^−^ CD16/32^Lo^ CD34^−^), and granulocyte/monocyte progenitor (GMP; Lin^−^ c–Kit^+^ Sca‐1^−^ CD16/32^Hi^ CD34^+^) cells were stained with biotin Ab cocktail, PerCP/Cy5.5‐streptavidin, APC‐c‐Kit Ab, PE/Cy7‐Sca‐1 Ab, PE‐CD34 Ab (BioLegend, Cat# 152203, RRID: AB_2629647), and APC/Cy7‐CD16/32 Ab (BioLegend, Cat# 101327, RRID: AB_1967102). Common lymphoid progenitor (CLP; IL–7R^+^ Lin^−^ c–Kit^+^ Sca‐1^−^) cells were stained with biotin Ab cocktail, PerCP/Cy5.5‐streptavidin, APC‐c‐Kit Ab, PE/Cy7‐Sca‐1 Ab, and PE‐IL7Ra Ab (BioLegend, Cat# 158203, RRID: AB_2876545). Peripheral blood was hemolyzed, treated with Fc blocker, and stained with APC/Cy7‐CD45 Ab (BD Biosciences, Cat# 561037, RRID: AB_10563075), FITC‐CD45.1 Ab (BioLegend, Cat# 110705, RRID: AB_313494), PacificBlue‐CD45.2 Ab (BioLegend, Cat# 109819, RRID: AB_492873), PE‐Gr‐1/Mac1 Ab (BioLegend, Cat# 108407, RRID:AB_313372), PE/Cy7‐CD3 Ab (BioLegend, Cat# 100219, RRID: AB_1732068), and APC‐CD19 Ab (BioLegend, Cat# 152409, RRID: AB_2629838). To analyze Notch1 and Notch2 expression, cultured cell suspensions were hemolyzed, treated with Fc blocker, and stained with biotin Ab, PerCP/Cy5.5‐streptavidin, APC‐c‐Kit Ab, and PE/Cy7‐Sca‐1 Ab with PE‐Notch Ab (BioLegend, Cat# 130607, RRID: AB_1227719), PE‐Notch2 Ab (BioLegend, Cat# 130707, RRID: AB_1227725), or PE‐isotype control (BioLegend, Cat# 400508, RRID: AB_326530). Stained cells were analyzed by flow cytometry (BD FACS Canto II; BD Biosciences) using FlowJo software (TreeStar, Ashland, OR, USA).

### 2.6 Immunohistochemistry

Five days after a single 5‐FU administration, in vivo staining with CD150‐PE Ab (BioLegend, Cat# 115903, RRID: AB_313682) (2 μg per mouse) was performed. Femurs and tibias were harvested, fixed in 4% paraformaldehyde, and dehydrated in 30% sucrose solution. Frozen bone sections were prepared using the Kawamoto method [15]. Sections were stained with Ter‐119‐FITC Ab (BioLegend, Cat# 116205, RRID: AB_313706). Images of the sections were acquired using an inverted microscope (Ti2‐E; Nikon, Tokyo, Japan) equipped with a CSU‐W1 SoRa confocal scanner unit (Yokogawa Electric Corporation, Tokyo, Japan) and a Plan Apochromat Lambda S 20× objective lens (NA 0.75; Nikon) or Plan Apochromat 40× objective lens (NA 0.95; Nikon).

### 2.7 Competitive Repopulation Assay

Aliquots of 500 HSCs isolated from C57BL/6SJL (CD45.1) mice were cocultured with or without RBCs for 1 week. The cultured cells and 1 × 10^6^ bone marrow cells from C57BL6/J (CD45.2) mice were transplanted into C57BL6/J (CD45.2) mice irradiated at the dose of 10 Gy. Peripheral blood was collected from the eyes at 2, 4, and 8 weeks after transplantation, and flow cytometry was performed. The proportions of CD45.1^+^ cells among CD45^+^ leukocytes, Gr1/Mac1^+^ granulocytes or macrophages, CD3^+^ T cells, and CD19^+^ B cells were calculated.

### 2.8 RNA Sequencing

HSCs and LSK cells with or without RBCs were cultured for 1 day. The cultured cell suspensions were hemolyzed with ACK lysis buffer and treated with Fc blocker. Cells were stained with APC/Cy7‐CD45 Ab, and CD45‐positive cells were sorted using an SH800 cell sorter (Sony); approximately 3000 cells were collected per sample. Total RNA was extracted using QIAzol lysis reagent (Qiagen, Germantown, MD, USA) and sequenced using the HiSeq 2500 platform (Illumina, San Diego, CA, USA). Fold changes between samples were analyzed by two‐tailed Student's *t* test (*p* < 0.05) using the Subio Platform and Subio Basic Plug‐in v1.20 (Subio Inc., London, UK). RNA‐sequencing (RNA‐seq) data were deposited in the National Center for Biotechnology Information Gene Expression Omnibus database under accession number GSE287700. On the basis of the obtained data, pre‐processing, principal component analysis (PCA), and differential gene expression analysis were performed using iDEP v2.01 (http://bioinformatics.sdstate.edu/idep/) [16].

Pathway analysis was performed using the PROGENy (Pathway RespOnsive GENes) footprint method (progeny v. 1.20) with the decoupleR package [17]. The voom‐normalized expression matrix was input into a multivariate linear model (MLM) with the mouse PROGENy model [18], using signed gene weights for 14 core signaling pathways. The resulting pathway activity scores (positive = activation; negative = inhibition) were scaled across samples and visualized by bar plots. Hes1 regulatory network analysis was performed by importing literature‐curated Hes1 transcriptional interactions in mouse (organism = 10 090) via OmniPathR [19]. Only stimulatory edges or inhibitory edges were retained, and each target gene was assigned a mode of regulation value of +1 or −1. Limma‐derived moderated *t*‐statistics for the contrast of interest (Exp vs. Ctrl) were then extracted for all Hes1 targets. Activating (mor = +1) and inhibiting (mor = −1) gene sets were each ranked by the absolute value of their *t*‐statistics, and bar plots of *t*‐values (sorted by |*t*|) were used to visualize the top‐ranked targets in each group. Pathway analysis and Hes1 regulatory network analysis were performed in R (v. 4.2.0) on MacOS.

### 2.9 Quantitative Real‐Time PCR

Total RNA and cDNA from HSCs were prepared using an RNeasy Mini Kit (QIAGEN, Hilden, Germany) and a high‐capacity RNA‐to‐cDNA kit (Thermo Fisher Scientific) following the manufacturers' instructions. Relative expression levels of Hes1 were evaluated according to the TaqMan gene expression assay protocol (Thermo Fisher Scientific). Quantitative real‐time PCR (q–PCR) was performed using QuantStudio7 (Thermo Fisher Scientific). Gene expression was calculated relative to the housekeeping gene *Gapdh*. Taqman FAM dye‐labeled MGB probe sets for *Notch1* (Mm00627185_m1), Notch2 (Mm00803077_m1), *Hes1* (Mm01342805_m1) and *Gapdh* (Mm99999915_g1) were purchased from Thermo Fisher Scientific.

### 2.10 Burst‐Forming Unit‐Erythroid Assay

CD48^−^ CD150^+^ LSK cells were isolated from HSCs that have been cultured with or without RBCs for 7 days. Aliquots of 5000 CD48^−^ CD150^+^ LSK cells were cultured for 10 days in medium with EPO (MethoCult SF M3436; STEMCELL Technologies, Vancouver, BC, Canada) using 3.5‐cm dishes at 37°C in 5% CO_2_. The numbers of burst‐forming unit‐erythroid (BFU‐E) colonies were counted.

### 2.11 Statistics and Reproducibility

Differences between two groups were analyzed using the two‐tailed unpaired Student's *t* test. Differences among three or more groups were evaluated using one‐way analysis of variance (ANOVA). Statistical analysis was performed using Prism v. 9 (GraphPad Software, La Jolla, CA, USA). The numbers of samples and animals included in these analyses are indicated in the figures.

## 3 Results

### 3.1 Stress–Induced Increase in RBCs Enhances Hematopoiesis

**FIGURE 1:**
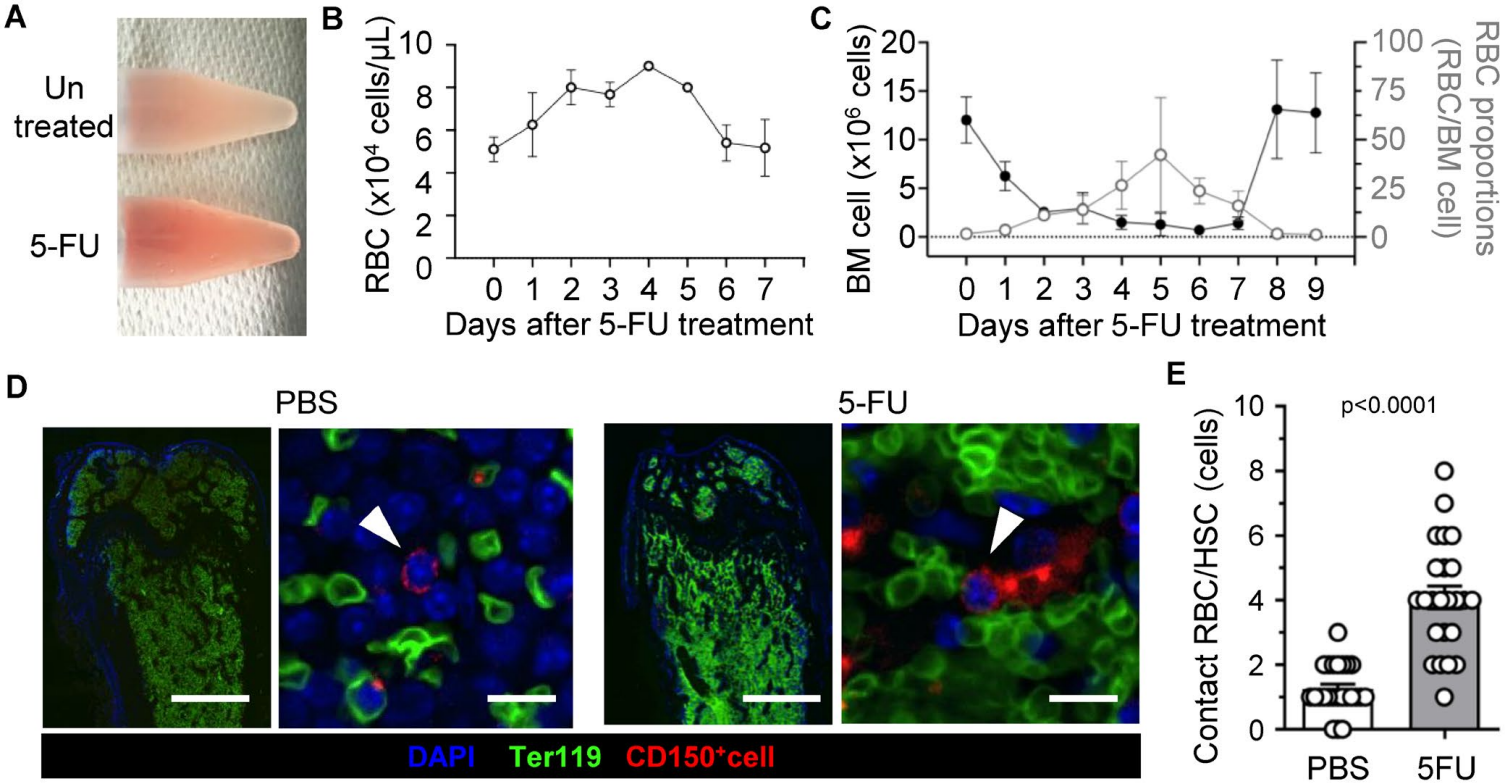
Red blood cells (RBCs) expand early in the recovery phase after 5‐FU treatment. (A) Images of bone marrow (BM) cell suspension before (Untreated) and 5 days after 5‐FU administration (5‐FU). (B) Trends in RBC numbers collected from BM during 5‐FU administration (*n* = 6 mice per group). (C) Trends in BM cell numbers and RBC proportions among cells collected from BM during 5‐FU administration (*n* = 3–10 mice per group). (D) Confocal microscopy images of frozen bone marrow sections from controls (PBS) and at 4 days after 5‐FU administration (5‐FU). Single nuclear CD150^+^ cells and nonnuclear Ter119^+^ cells (RBCs) were detected at low (left panel) and high magnification (right panel). Green, Ter119; red, CD150; blue, DAPI. Scale bars = 1 mm (left panel) and 10 μm (right panel). (E) Quantification of numbers of RBCs in contact with a single CD150^+^ cell (*n* = 24 per group). All data are presented as means ± SD. *P*‐values are shown in the figures.

Bone marrow conditions are markedly altered by chemotherapy. We analyzed bone marrow dynamics over time to investigate the recovery phase following 5‐FU administration. On day 5 post‐administration, collected bone marrow cells turned red, implying the presence of RBCs (Figure 1A). The RBC concentration peaked on day 5 and then decreased and returned to the baseline; hematopoiesis recovered by day 8 (Figure 1B). The RBC concentration in peripheral blood peaked on day 4 and then decreased to the baseline by day 6 (Figure 1C). To visualize the spatial relationship between HSCs—the primary drivers of hematopoiesis—and RBCs, we performed immunohistological analysis of bone marrow sections. Compared to untreated controls, Ter119^+^ RBC density was markedly higher following 5‐FU administration (Figure 1D), and the number of RBCs making direct contact with CD150^+^ HSCs increased by approximately fourfold under 5‐FU treatment (Figure 1D,E).

**FIGURE 2:**
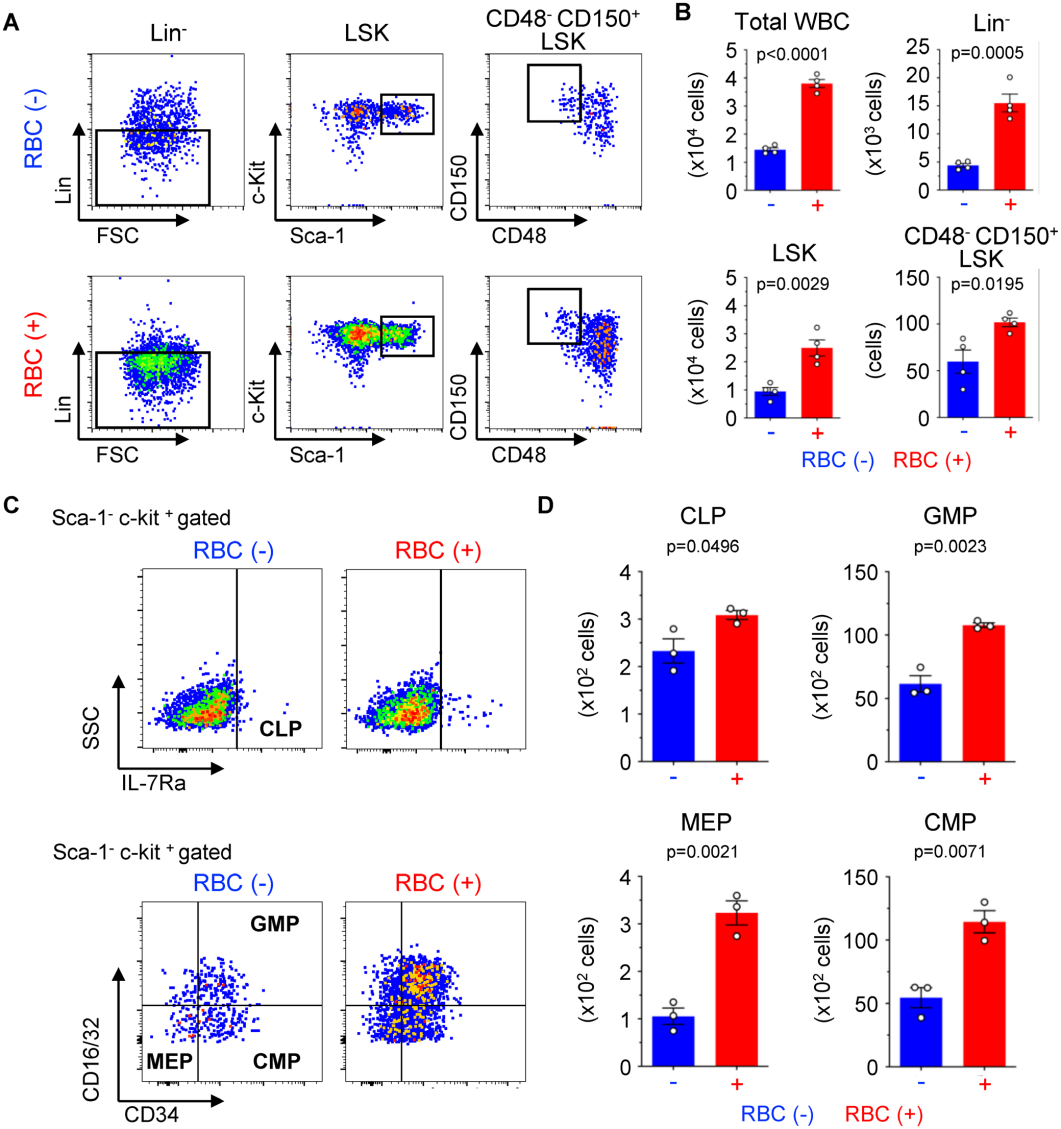
RBCs stimulate the expansion of hematopoietic cells. (A, B) Total white blood cells (WBCs), lineage‐negative (Lin^−^) cells, LSK cells, and CD48^−^ CD150^+^ LSK cells; and (C, D) progenitor cells after culture of 500 HSCs with or without 1.0 × 10^7^ RBCs for 7 days. (A) Flow cytometric analysis of Lin^−^, LSK, and CD48^−^ CD150^+^ LSK cell populations. (B) Numbers of WBCs, Lin^−^ cells, LSK cells, and CD48^−^ CD150^+^ LSK cells (*n* = 4 per group). (C) Flow cytometric analysis of common lymphoid progenitor (CLP), common myeloid progenitor (CMP), granulocyte/monocyte progenitor (GMP), and megakaryocyte/erythrocyte progenitor (MEP) populations. (D) Numbers of CLP, CMP, GMP, and MEP cells (*n* = 3 per group). All data are presented as means ± SD. *P*‐values are shown in the figures.

To assess the effects of RBCs on HSC function, we isolated CD48^−^ CD150^+^ LSK cells as HSCs, which maintain permanent hematopoiesis [20], and cultured them with or without RBCs. Coculture with RBCs led to significant increases in the numbers of total white blood cells (WBCs), Lin^−^ cells, LSK (Lin^−^ Sca‐1^+^ c–Kit^+^) cells, and CD48^−^ CD150^+^ LSK cells (Figure 2A,B). The observed increases were approximately 2.63‐fold for WBCs, 3.53‐fold for Lin^−^ cells, and 1.71–fold for CD48^−^ CD150^+^ LSK cells (Figure 2B). Notably, the increase was more pronounced in hematopoietic progenitors, Lin^−^ cells and LSK cells, which had differentiated from the HSCs. We also investigated the amplification of myeloid progenitor fractions derived from the expanded HSCs. The different progenitor cell fractions (CLPs, MEPs, CMPs, and GMPs) were higher when cocultured with RBCs (Figure 2C,D). The increases were 1.32‐fold for CLPs, 3.07‐fold for MEPs, 2.10‐fold for CMPs, and 1.75‐fold for GMPs (Figure 2D). The increase was more pronounced for myeloid progenitors than for lymphoid progenitors. These findings imply that coculture of RBCs with HSCs can enhance the proliferation of hematopoietic progenitors and myeloid‐differentiated blood cells.

### 3.2 RBC‐Induced Enhancement of Hematopoiesis Requires Direct Cell–Cell Contact

**FIGURE 3:**
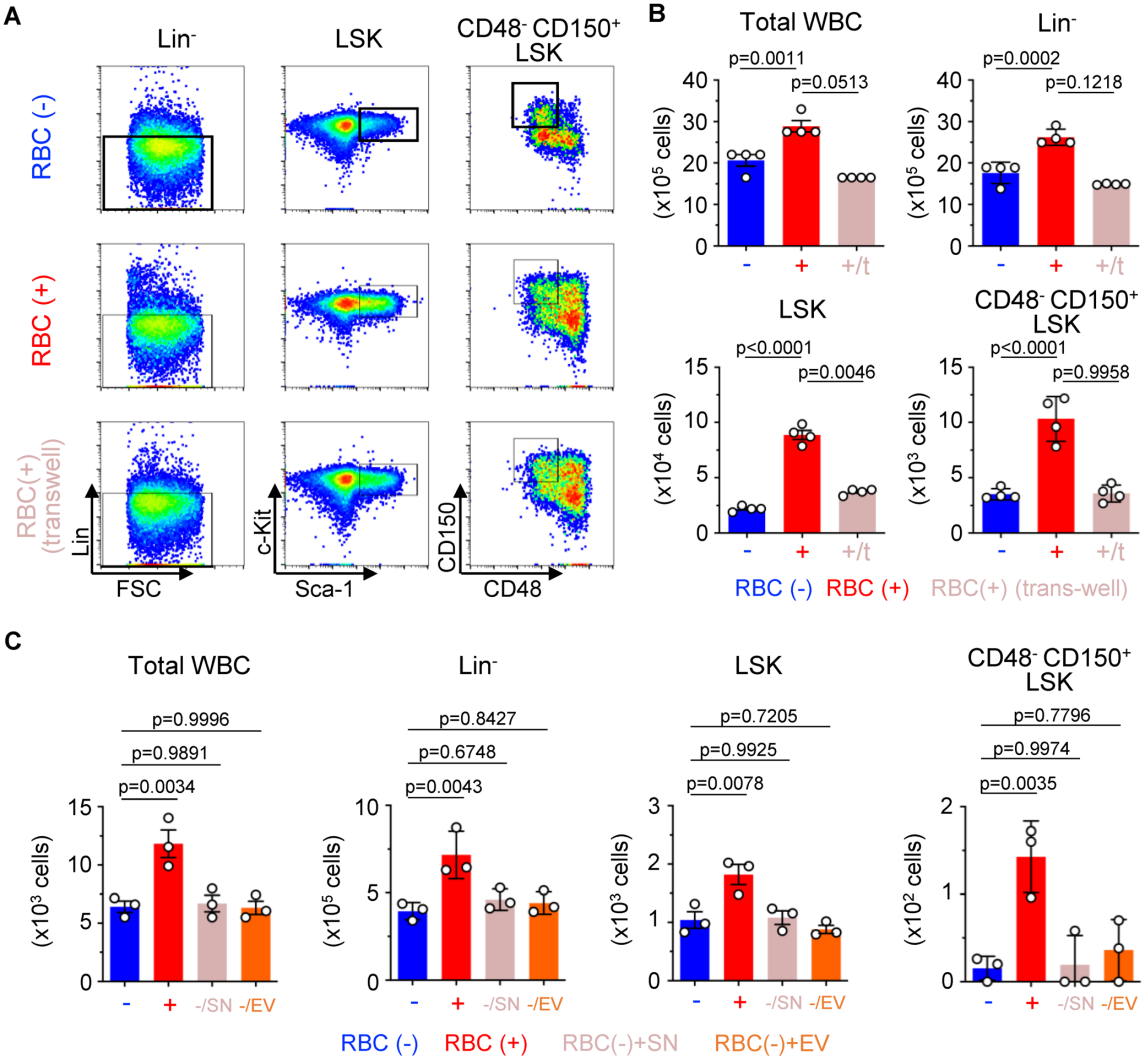
RBC‐induced hematopoiesis requires direct cell contact. (A, B) Progenitor cells after culture of 1000 HSCs with or without 5.0 × 10^6^ RBCs or in a Transwell system for 7 days. (A) Flow cytometric analysis of Lin^−^ cells, LSK cells, and CD48^−^ CD150^+^ LSK cells. (B) Numbers of WBCs, Lin^−^ cells, LSK cells, and CD48^−^ CD150^+^ LSK cells are shown (*n* = 4 per group). (C) Analysis of progenitor cells after culture of 500 HSCs with supernatant (SN), extracellular vesicles (EVs), or 1.0 × 10^7^ RBCs for 7 days. Numbers of WBCs, Lin^−^ cells, LSK cells, and CD48^−^ CD150^+^ LSK cells are shown (*n* = 3 per group). All data are presented as means ± SD. *P*‐values are shown in the figures.

To determine whether direct contact with RBCs is essential for the enhancement of hematopoiesis, we utilized a Transwell coculture system that allows the exchange of soluble factors but prevents direct cell–cell interaction. The increases in WBCs, Lin^−^ cells, LSK cells, and CD48^−^ CD150^+^ LSK cells detected on coculture with RBCs were abolished when cells were separated by the Transwell membrane (Figure 3A,B). To examine the role of RBC‐derived factors further, we treated HSCs with either EV or SN collected from cultured RBCs. Neither RBC‐derived EV nor SN replicated the proliferative effect seen in direct coculture (Figure 3C), reinforcing the conclusion that soluble factors alone are insufficient and that direct RBC–HSC contact is critical for enhanced cellular proliferation. We next examined whether this effect could be mimicked by physical clustering with inert particles of similar size to RBCs. HSCs were cocultured with polystyrene microbeads 5 μm in diameter, chosen to approximate the dimensions of RBCs. However, flow cytometric analysis showed that the presence of the microbeads failed to enhance hematopoiesis (Figure S1). These findings excluded the possibility that cell proliferation is driven simply by nonspecific physical proximity to similarly sized objects.

These in vitro results were consistent with our in vivo observations following 5‐FU treatment that increased physical proximity between RBCs and HSCs in the bone marrow coincided with enhanced hematopoiesis (Figure 1D,E). Taken together, our data show that direct interaction with RBCs, and not exposure to soluble factors or size‐matched mimics, is required to stimulate enhancement of hematopoiesis in HSCs.

### 3.3 RBC‐Induced Hematopoietic Enhancement Is Prominent Early in Bone Marrow Recovery In Vivo

**FIGURE 4:**
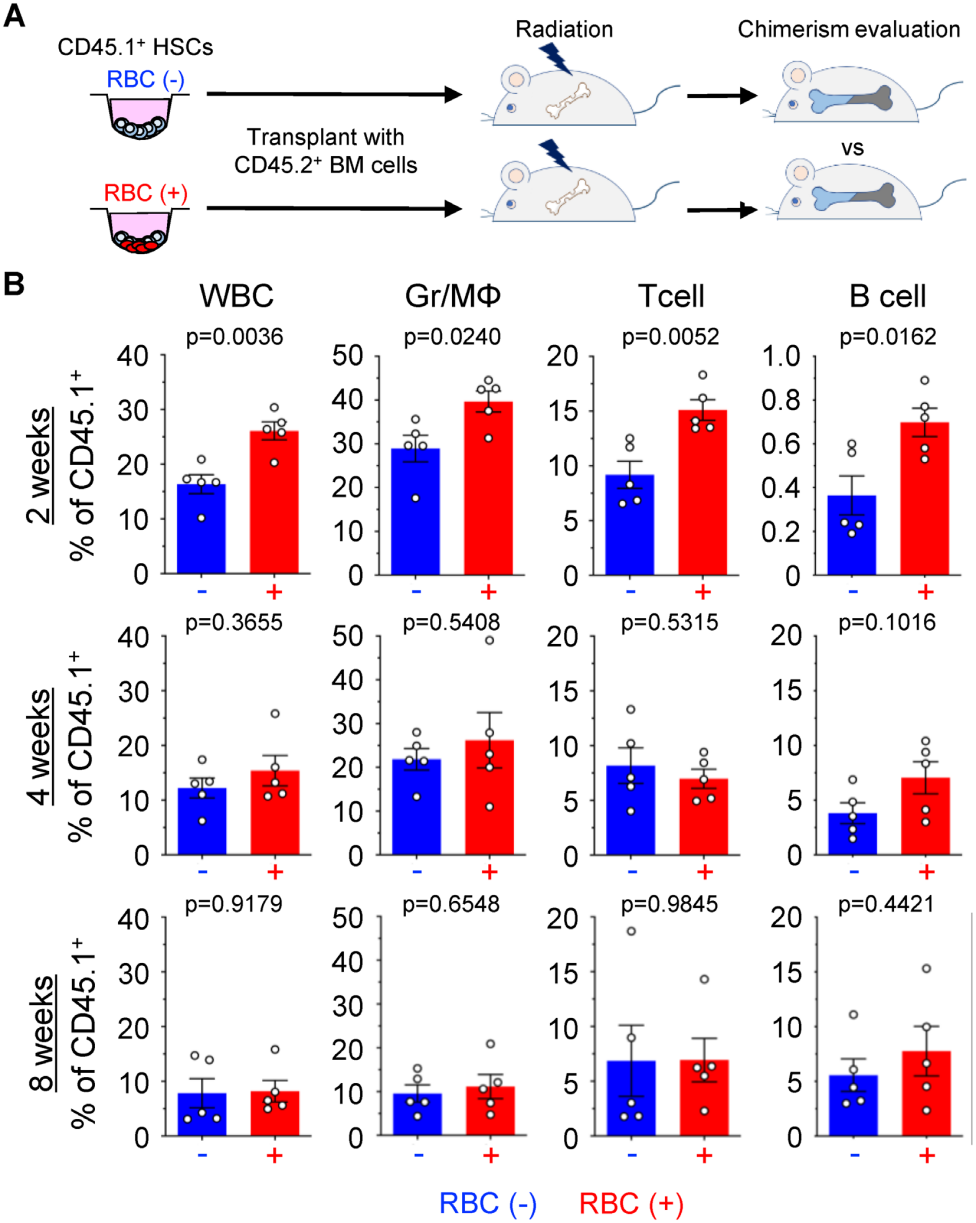
RBCs induced hematopoiesis in vivo. (A) Protocol for the competitive transplantation assay. Samples of 500 HSCs from donor mice (CD45.1^+^ mice) with or without 1.0 × 10^7^ RBCs were cultured and transplanted into irradiated recipient mice (CD45.2^+^ mice). (B) Evaluation of chimerism at 2, 4, and 8 weeks by competitive transplantation assay (*n* = 5 per group). All data are presented as means ± SD. *P*‐values are shown in the figures.

To determine whether HSCs cocultured with RBCs enhance hematopoietic cell amplification in vivo, a competitive repopulation assay was conducted (Figure 4A). CD45.1^+^ HSCs, precultured with or without RBCs, were transplanted into irradiated CD45.2^+^ congenic recipients, and the hematopoietic responses of donor cells were investigated. At 2 weeks after transplantation, the proportions of donor‐derived mature hematopoietic cell populations (WBCs, granulocyte/macrophage lineage cells, T cells, and B cells) were significantly higher in recipients that were transplanted with HSCs cocultured with RBCs (Figure 4B), confirming the enhancement of hematopoietic function in vivo. However, these differences were marginal at 4 and 8 weeks after transplantation (Figure 4B). These findings indicate that RBCs promote the short‐term hematopoietic activity of HSCs, likely by supporting the generation of short‐term hematopoietic stem cells (ST‐HSCs) and multipotent progenitors (MPPs). This leads to the transient expansion of hematopoietic progenitors contributing to both myeloid and lymphoid lineages during bone marrow recovery.

### 3.4 Transcription Factors Linked to RBC–Mediated Modulation of Hematopoiesis

**FIGURE 5:**
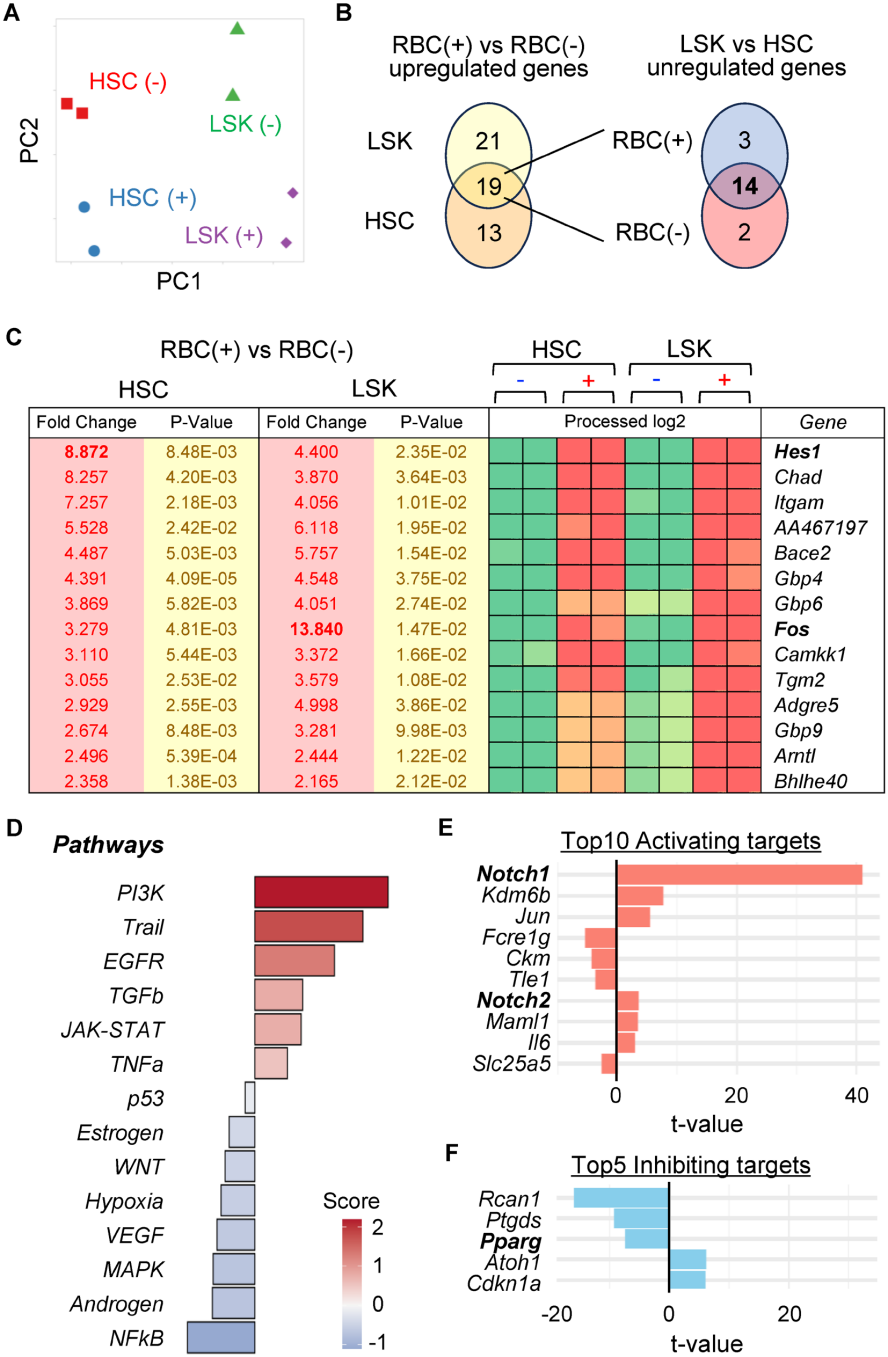
Bioinformatics analysis of HSCs and LSK cells cocultured with RBCs. (A) Principal component analysis (PCA) plot of bulk RNA‐seq data. (B) Venn diagrams were generated using the limma trend method for differential gene expression analysis. Genes highly upregulated in both HSCs and LSK cells cultured with RBCs are indicated in bold. (C) Comparative analysis of the 14 genes indicated in bold in (B). (D) Pathway enrichment analysis using the PROGENy method in LSK cells cocultured with RBCs. (E, F) Network analysis using the OmniPath database in LSK cells cocultured with RBCs. Expression changes of known Hes1‐regulated top 10 activating targets (E) and top 5 inhibiting targets (F) are shown.

To identify the factors responsible for the RBC‐induced enhancement of the hematopoietic function of HSCs, we conducted RNA‐seq of HSCs and LSK cells cultured in the presence or absence of RBCs. PCA revealed tight clustering across replicates, indicating that HSCs and LSK cells, as well as the presence or absence of RBCs, constituted distinct and reproducible subsets (Figure 5A). Differential gene expression analysis identified 40 upregulated genes in HSCs and 32 in LSK cells cocultured with RBCs; 19 genes were upregulated in both populations. Among these, 14 genes (*Hes1, Chad, Itgam, AA467197, Bace2, Gbp4, Gbp6, Fos, Camkk1, Tgm2, Adgre5, Gbp9, Arntl*, and *Bhlhe40*) exhibited comparable expression levels in HSCs and LSK cells (Figure 5B). *Hes1* was the transcription factor with the highest expression fold change in HSCs cocultured with RBCs (Figure 5C). *Hes1* maintains stem/progenitor cells by regulating cellular quiescence and fate decisions [21, 22]. Although *Hes1* is dispensable for steady‐state hematopoiesis, it plays key roles in maintaining stem/progenitor cells and protecting HSCs from replicative stress that can impair hematopoietic capacity [22]. To explore the pathways involved in RBC‐induced hematopoietic enhancement, we conducted pathway enrichment analysis using the PROGENy method, which predicts pathway activity based on gene expression signatures. The top enriched pathways in LSK cells cocultured with RBCs were PI3K, EGFR, JAK–STAT, and TNFα, all of which are associated with stem cell self‐renewal (Figure 5D). These results were consistent with our in vivo and in vitro findings that showed RBCs enhanced hematopoiesis. Notably, *Hes1* is a well‐established regulator of stem cell quiescence and fate decisions, further supporting its functional involvement in this context.

To elucidate downstream targets of *Hes1* further, we analyzed changes in the expression of known Hes1‐regulated genes in LSK cells cocultured with RBCs using the OmniPath database. Several key activators were upregulated, including *Notch1, Kdm6b, Jun, Notch2, Maml1*, *and Il6*, many of which have been implicated in HSC proliferation (Figures 5E, S2). Of these, *Notch1, but* not *Notch2*, is recognized as a central regulator of stem cell maintenance and differentiation [23]. Importantly, *Hes1* is also a direct target of Notch signaling, consistent with previous reports describing a positive feedback loop between Notch1 and Hes1 [24]. In addition, we identified *Pparg* as a gene that was suppressed in response to RBC exposure (Figures 5F, S2). As *Hes1* has been reported to mitigate replicative stress in HSCs via downregulation of PPARγ signaling [22], this finding provides further mechanistic support.

Taken together, these data support a model in which direct contact with RBCs activates the Notch1–Hes1 signaling axis, promoting self‐renewal and stress resilience in HSCs during hematopoietic recovery.

### 3.5 The Notch1–Hes1 Signaling Axis Influences RBC‐Mediated Modulation of Hematopoiesis

**FIGURE 6:**
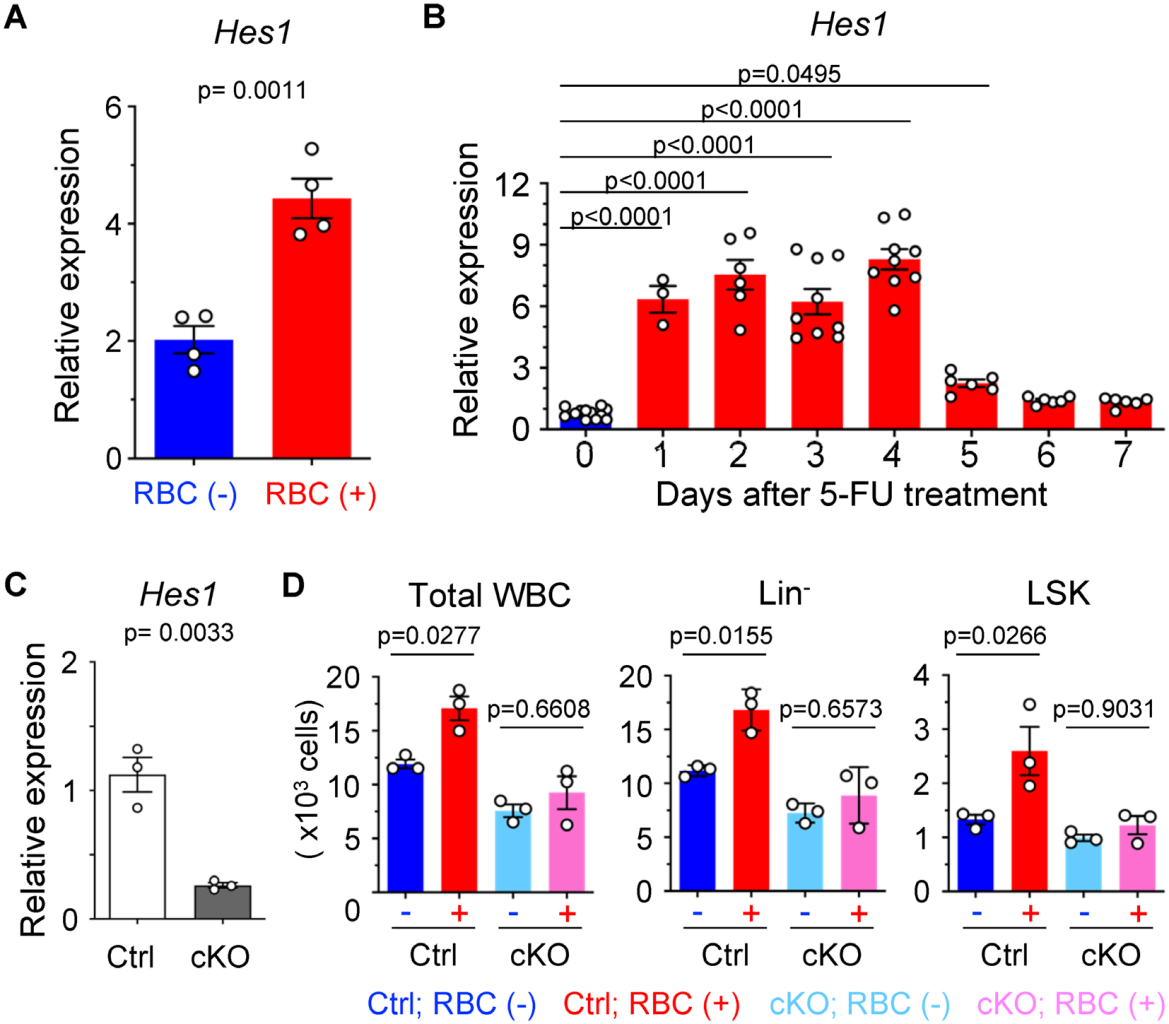
Hes1 modulates RBCs‐induced hematopoiesis. (A) *Hes1* expression levels in HSCs after culture with or without RBCs for 1 day (*n* = 4 per group). (B) *Hes1* expression levels in HSCs during 5‐FU administration (*n* = 3–12 per group). (C) Comparison of *Hes1* expression in HSCs collected from *Hes1* ^fl/fl^ (control, Ctrl) and *Hes1* ^flox/flox^; Vav1‐iCre (conditional knockout, cKO) mice. (D) Numbers of WBCs, Lin^−^ cells, LSK cells after culture of 500 HSCs with or without 1.0 × 10^7^ RBCs in Ctrl or cKO mice for 7 days (*n* = 3 per group). All data are presented as means ± SD. *P*‐values are shown in the figures.

Among the genes associated with RBC‐induced hematopoiesis, we focused on Hes1, which is implicated in replicative stress caused by transplantation [21, 22]. Hes1 expression was significantly increased in HSCs cocultured with RBCs (Figure 6A). Following a bone marrow injury by 5‐FU, we used qPCR to measure *Hes1* gene expression in HSCs. *Hes1* expression increased significantly from day 1 to day 5 after single administration (Figure 6B), coinciding with the increase in RBC concentrations (Figure 1B). To evaluate the role of *Hes1* in RBC‐induced hematopoiesis, we generated hematopoietic lineage‐specific Hes1 conditional knockout mice (*Hes1* ^flox/flox^; Vav1‐iCre). HSCs isolated from these mice had an approximately 4.3‐fold reduction in Hes1 expression (Figure 6C). Coculturing Hes1 knockout HSCs with RBCs reversed the increases in WBCs, Lin^−^ cells, LSK cells, and CD48^−^ CD150^+^ LSK cells in the control (Figure 6D). These findings show that Hes1 is a key mediator of RBC‐induced hematopoietic activation.

**FIGURE 7:**
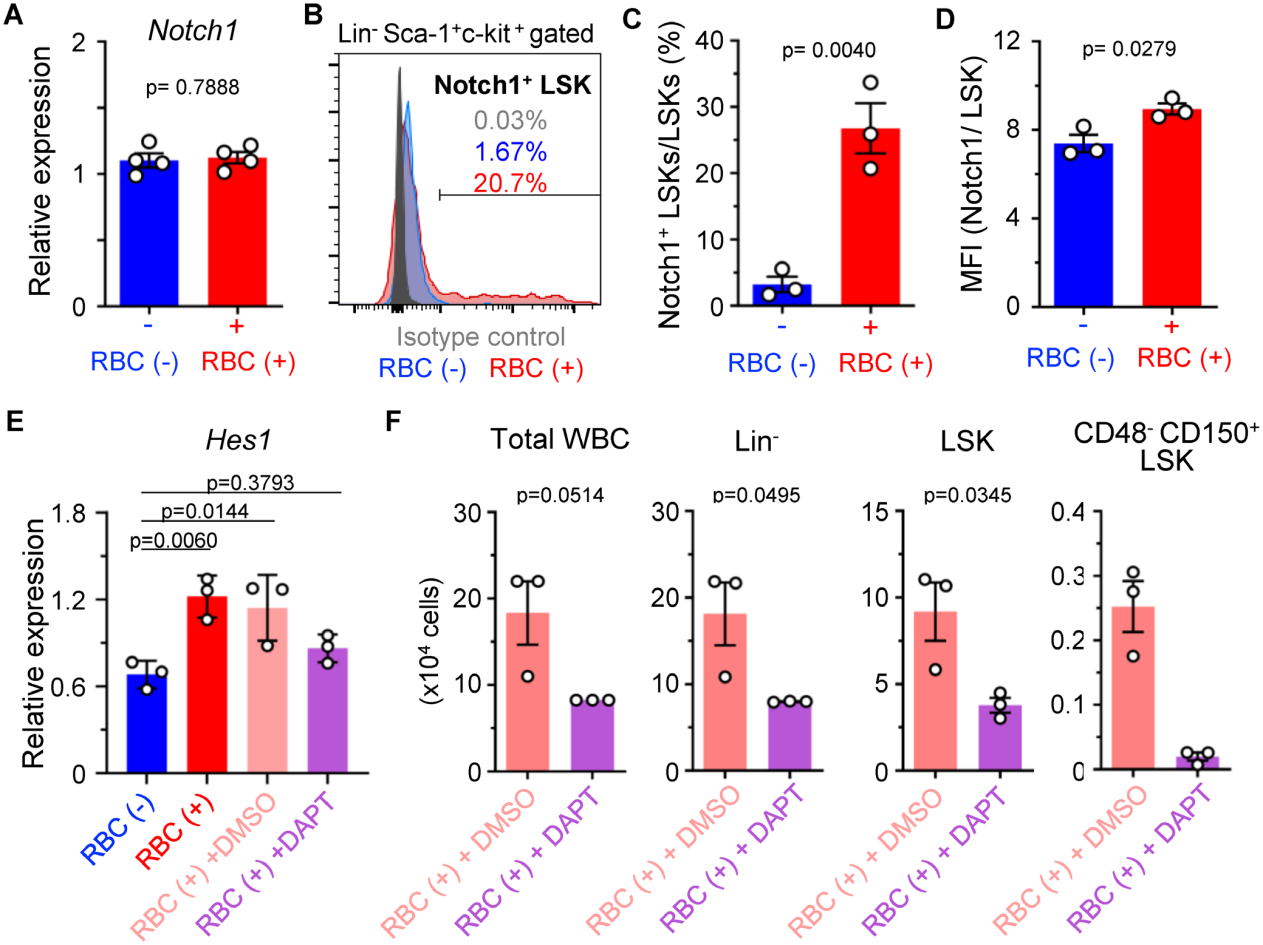
Notch1 modulates RBC‐induced hematopoiesis via Hes1. (A) Notch1 expression levels in HSCs after culture with or without RBCs for 1 day (*n* = 4 per group). (B–D) Flow cytometric analysis of LSK cells after culture of 500 HSCs with or without 1.0 × 10^7^ RBCs for 1 day. (B) Histogram of LSK cells. (C) The percentages of Notch1^+^ LSK cells among LSK cells are shown (*n* = 3 per group). (D) Median fluorescence intensity (MFI) (*n* = 3 per group). (E) *Hes1* expression levels in HSCs after culture of 500 HSCs with 1.0 × 10^7^ RBCs and solvent control (DMSO) or 10 μM DAPT for 1 day (*n* = 3 per group). (F) Analysis of progenitor cells after culture of 500 HSCs with 1.0 × 10^7^ RBCs and DMSO or 10 μM DAPT for 4 days. Numbers of WBCs, Lin^−^ cells, LSK cells, and CD48^−^ CD150^+^ LSK cells are shown (*n* = 3 per group). All data are presented as mean ± SD. *P*‐values are shown in the figures.

As noted above, Notch1 and Notch2 were detected among Hes1‐associated targets, and *Hes1* itself is a direct downstream target of Notch signaling, implying a potential positive feedback loop. To explore the role of Notch signaling under conditions of hematopoietic stress further, we evaluated Notch1 and Notch2 expression in HSCs. qPCR analysis revealed no significant changes in *Notch1* or *Notch2* mRNA levels with or without RBCs (Figure 7A, Figure S3A). However, flow cytometric analysis revealed that both the frequency of the Notch1^+^ population and the median fluorescence intensity (MFI) of Notch1^+^ in LSK cells increased significantly upon RBC coculture by approximately 7.08‐fold and 1.71‐fold, respectively (Figure 7B–D), while Notch2 expression remained unchanged (Figure S3B–D).

To evaluate the functional relationship between Notch1 and Hes1, we examined *Hes1* expression in HSCs cultured with RBCs in the presence or absence of the Notch1 inhibitor DAPT. While DMSO‐treated controls maintained elevated *Hes1* expression levels in the presence of RBCs, the addition of DAPT reduced *Hes1* expression to levels comparable to those observed in cultures without RBCs (Figure 7E). Furthermore, the addition of DAPT abolished the RBC‐induced increases in total WBCs, Lin^−^ cells, LSK cells, and CD48^−^ CD150^+^ LSK cells (Figure 7F), confirming that Notch1 activity is essential for the hematopoietic effects mediated by RBCs.

Taken together, these findings show that Notch1, but not Notch2, plays a critical role in mediating the *Hes1*‐dependent response to RBCs. This establishes the Notch1–Hes1 signaling axis as a central mechanism by which RBCs enhance HSC function during stress‐induced hematopoietic recovery.

## 4 Discussion

Our findings provide insights into the relationship between RBCs and HSC function during bone marrow recovery, particularly in the context of chemotherapy‐induced stress. The key findings implicate RBCs in the proliferation and differentiation of HSCs and indicate that this effect is mediated by direct cell–cell contact via the Notch1–Hes1 signaling axis.

RBC concentrations in bone marrow increased transiently after 5‐FU administration, coinciding with a peak in hematopoietic progenitor cell proliferation. This result implies that RBCs contribute to the modulation of hematopoiesis in response to stress‐induced bone marrow damage. The proportion of RBCs relative to HSCs increased up to tenfold during the recovery phase following 5‐FU administration compared to steady‐state conditions. This increase could not be explained solely by a temporary decrease in WBCs due to the cytotoxicity of 5‐FU to rapidly dividing cells. Our data indicate that erythropoiesis is upregulated in response to bone marrow suppression. Supporting this finding, the RBC/HSC ratio in bone marrow and the RBC concentration in peripheral blood increased early in the recovery phase, implying that erythropoiesis is activated in bone marrow, enhancing RBC release into the circulatory system. This response is likely driven by increased erythropoietin (EPO) secretion in response to 5‐FU‐induced anemia [25, 26, 27, 28]. In addition, the BFU‐E colony‐forming ability of HSCs cocultured with RBCs was not enhanced (Figure S4), and the EPO receptor was not upregulated in both HSCs and LSK cells cocultured with RBCs. These results imply that the sensitivity of HSCs to EPO was unchanged, indicating that the RBC increase was primarily a result of enhanced differentiation caused by an elevated EPO level. Collectively, these findings support the notion that the transient increase in RBCs is not a consequence of chemotherapy‐induced stress but is a functionally relevant adaptation driven by intrinsic regulatory mechanisms.

In vitro coculture showed that direct RBC–HSC interactions significantly enhance the proliferation of HSCs and their downstream progenitor populations. This effect was absent in a Transwell system, indicating that direct cell–cell contact is essential for RBC‐mediated enhancement of hematopoiesis. Furthermore, RBC‐derived EVs and soluble factors alone or size‐matched mimics had no effect, reinforcing the importance of direct contact between RBCs and HSCs. Our finding highlights a previously unrecognized role of RBCs in modulating hematopoiesis. Although formerly considered passive oxygen carriers, RBCs have diverse functions, including miRNA‐mediated intercellular signaling and regulation of inflammatory and immune responses [29, 30, 31]. In particular, RBCs contain high levels of miRNAs, which are secreted via EVs; some (e.g., miR‐451, miR‐144, miR‐486, and miR‐4732‐3p) promote erythroid differentiation [32, 33, 34, 35]. However, EV supplementation did not significantly influence the hematopoietic potential of HSCs after 5‐FU administration, indicating that the RBC‐mediated enhancement of HSC function is not driven by miRNAs in EVs. RBCs regulate immune responses by binding to chemokines as scavengers at inflammatory sites and releasing them into plasma when chemokine levels decline, thereby maintaining homeostasis [36]. The chemokine stromal cell‐derived factor 1 (SDF‐1) binds to the Duffy antigen receptor for chemokines (DARC) expressed on RBCs and their precursors [37]. SDF‐1 enhances the synergistic effects of IL‐6, IL‐12, and stem cell factor (SCF) on the proliferation of HSCs, potentially protecting quiescent cells from 5‐FU–induced damage [38, 39]. This regulatory role of RBCs implies a potential mechanism by which RBCs contribute to the restoration of bone marrow homeostasis following chemotherapy‐induced suppression.

Using transcriptomic profiling, we identified Hes1 as the key transcription factor in RBC‐mediated enhancement of hematopoiesis. Hes1, a bHLH transcriptional repressor, is highly expressed in quiescent HSCs and maintains stem/progenitor cells by inhibiting cell cycling and expansion while preserving long‐term reconstitution activity [21, 40]. Functional analyses using *Hes1* conditional knockout mice showed that Hes1 is essential for RBC‐mediated enhancement of hematopoiesis. Given that Hes1 regulates stem/progenitor cell maintenance under transplant stress [22], our findings imply that RBCs support hematopoietic recovery following chemotherapy‐induced damage via activation of Hes1‐dependent pathways. Supporting this model, pathway enrichment analysis revealed activation of the PI3K, EGFR, JAK–STAT, and TNFα signaling pathways, each of which supports stem cell maintenance and proliferation, thus indicating that RBCs initiate a broad transcriptional program to enhance hematopoietic regeneration. Importantly, our data demonstrated that this effect is mediated via Notch1, as evidenced by increased surface expression of Notch1 following RBC coculture and the loss of RBC‐induced hematopoietic effects upon Notch1 inhibition. Although Notch1 mRNA levels remained unchanged, the elevated protein expression implies that RBCs enhance Notch1 signaling at the posttranscriptional level, leading to downstream activation of Hes1 and its associated target genes. The essential role of Hes1 was further confirmed using Hes1‐deficient mice; deletion of *Hes1* abrogated the RBC‐induced increases in HSCs. Similarly, pharmacological inhibition of Notch1 signaling using DAPT phenocopied the effects of *Hes1* deletion, reinforcing the conclusion that RBC‐mediated hematopoietic enhancement is critically dependent on Notch1‐driven *Hes1* expression.

Taken together, our findings reveal that RBCs play a previously unrecognized role in modulating HSC function through the Notch1–Hes1 signaling axis. This mechanism is a novel physiological function of RBCs other than oxygen transport and likely represents an adaptive response to hematopoietic stress, facilitating rapid regeneration of bone marrow. Understanding the interplay between RBCs and HSCs could facilitate improvement of hematopoietic recovery in clinical settings, such as after chemotherapy or bone marrow transplantation.

## Author Contributions

T.S. and M.I. conceived the study; E.Y. and T.S. designed the experiments; E.Y., S.H., H.A., and T.O. performed the experiments with the assistance of T.S.; E.Y. and S.H. performed data analysis with the assistance of T.S.; H.A. and D.O. contributed to the sequencing analyses. E.Y., T.S., and M.I. wrote the manuscript.

## Disclosure

The authors have nothing to report.

## Conflicts of Interest

The authors declare no conflicts of interest.

## Supporting information


**Data S1:** fsb271022‐sup‐0001‐Supinfo.docx.

**Figure S1:** Size‐matched RBC mimics failed to induce hematopoiesis. Analysis of progenitor cells after culture of 500 HSCs with 1.0 × 10^7^ of RBCs or 5‐μm microbeads. Numbers of WBCs, Lin^−^ cells, LSK cells, and CD48^−^ CD150^+^ LSK cells are shown (*n* = 4 per group). All data are presented as means ± SD. *P*‐values are shown in the figure.
**Figure S2:** Differential expressions of Hes1‐responsive genes was evaluated. Hes1 regulatory network analysis using the OmniPath database of LSK cells cocultured with RBCs. Whole Hes1‐regulated targets are shown. Red, activating targets; blue, inhibiting targets.
**Figure S3:** Notch2 is not related to RBC‐induced hematopoiesis. (A) Notch2 expression levels in HSCs after culture with or without RBCs for 1 day (*n* = 4 per group). (B–D) Flow cytometric analysis of LSK cells after culture of 500 HSCs with or without 1.0 × 10^7^ RBCs for 1 day. (B) Histogram of LSK cells. (C) The percentages of Notch2^+^ LSK cells among LSK cells are shown (*n* = 3 per group). (D) Median fluorescence intensity (MFI) of Notch2 (*n* = 3 per group). All data are presented as means ± SD. *P*‐values are shown in the figures.
**Figure S4:** The BFU‐E colony‐forming ability of HSCs cocultured with RBCs was not enhanced. Comparison of the BFU‐E colony forming by CD48^−^ CD150^+^ LSK cells isolated from HSCs cultured with or without RBCs. Numbers of BFU‐E colonies after 10 days of culture in MethoCult (medium with EPO) are shown (*n* = 6 per group). All data are presented as means ± SD. *P*‐value is shown in the figure.

## Data Availability

The data that support the findings of this study are available in the Materials and Methods, Results, and/or Supporting Information of this article.
