# Supplementary material for: Red Blood Cell‐Mediated Enhancement of Hematopoietic Stem Cell Functions via a Hes1‐Dependent Pathway
Source: FASEB J. 2025 Sep 9;39(17):e71022. doi: 10.1096/fj.202500885R (PMC12418148; doi:10.1096/fj.202500885R)
Supplement: Supplementary file 1 — Data S1: fsb271022‐sup‐0001‐Supinfo.docx. [file FSB2-39-e71022-s001.docx]

**Figure legend only for reviewer**

Flow cytometry analysis of RBCs.

RBCs were stained with PE-Dll1 Ab (BioLegend, Cat# 128307, RRID: AB_1133995), PE-Dll4 Ab (BioLegend, Cat# 130807, RRID: AB_1227634), PE-CD339 (Jagged1) Ab (BioLegend Cat# 130907, RRID: AB_2561302), PE-Jagged2 Ab (BioLegend Cat# 131007, RRID: AB_2128358) or PE-isotype control. (A, B) Histogram plot of HSCs. (C, D) Median fluorescent intensity (MFI) of RBCs expressing Dll1, Dll4, Jag1 or Jag2 (*n* = 4 per group). All data are presented as means ± SD. *P*-values are shown in the figures.
