# Supplementary material for: Red Blood Cell‐Mediated Enhancement of Hematopoietic Stem Cell Functions via a Hes1‐Dependent Pathway
Source: FASEB J. 2025 Sep 9;39(17):e71022. doi: 10.1096/fj.202500885R (PMC12418148; doi:10.1096/fj.202500885R)
Supplement: Supplementary file 2 — Figure S1: Size‐matched RBC mimics failed to induce hematopoiesis. Analysis of progenitor cells after culture of 500 HSCs with 1.0 × 10^7^ of RBCs or 5‐μm microbeads. Numbers of WBCs, Lin− cells, LSK cells, and CD48− CD150+ LSK cells are shown (n = 4 per group). All data are presented as means ± SD. P‐values are shown in the figure. Figure S2: Differential expressions of Hes1‐responsive genes was evaluated. Hes1 regulatory network analysis using the OmniPath database of LSK cells cocultured with RBCs. Whole Hes1‐regulated targets are shown. Red, activating targets; blue, inhibiting targets. Figure S3: Notch2 is not related to RBC‐induced hematopoiesis. (A) Notch2 expression levels in HSCs after culture with or without RBCs for 1 day (n = 4 per group). (B–D) Flow cytometric analysis of LSK cells after culture of 500 HSCs with or without 1.0 × 10^7^ RBCs for 1 day. (B) Histogram of LSK cells. (C) The percentages of Notch2+ LSK cells among LSK cells are shown (n = 3 per group). (D) Median fluorescence intensity (MFI) of Notch2 (n = 3 per group). All data are presented as means ± SD. P‐values are shown in the figures. Figure S4: The BFU‐E colony‐forming ability of HSCs cocultured with RBCs was not enhanced. Comparison of the BFU‐E colony forming by CD48− CD150+ LSK cells isolated from HSCs cultured with or without RBCs. Numbers of BFU‐E colonies after 10 days of culture in MethoCult (medium with EPO) are shown (n = 6 per group). All data are presented as means ± SD. P‐value is shown in the figure. [file FSB2-39-e71022-s002.zip › 202500885R-sup-0003-SI_Text-S01.docx]

**Supplemental file**

**Supplemental Figure 1.** **Size-matched RBC mimics failed to induce hematopoiesis.**

Analysis of progenitor cells after culture of 500 HSCs with 1.0 × 10^7^ of RBCs or 5-μm microbeads. Numbers of WBCs, Lin^−^ cells, LSK cells, and CD48^−^ CD150^+^ LSK cells are shown (*n* = 4 per group). All data are presented as means ± SD. *P*-values are shown in the figure.

**Supplemental Figure 2. Differential expressions of Hes1-responsive genes was evaluated.**

Hes1 regulatory network analysis using the OmniPath database of LSK cells cocultured with RBCs. Whole Hes1-regulated targets are shown. Red, activating targets; blue, inhibiting targets.

**Supplemental Figure 3. Notch2 is not related to RBC-induced hematopoiesis.**

(A) Notch2 expression levels in HSCs after culture with or without RBCs for 1 day (*n* = 4 per group). (B–D) Flow cytometric analysis of LSK cells after culture of 500 HSCs with or without 1.0 × 10^7^ RBCs for 1 day. (B) Histogram of LSK cells. (C) The percentages of Notch2^+^ LSK cells among LSK cells are shown (*n* = 3 per group). (D) Median fluorescence intensity (MFI) of Notch2 (*n* = 3 per group). All data are presented as means ± SD. *P*-values are shown in the figures.

**Supplemental Figure 4. The BFU-E colony-forming** **ability of HSCs cocultured with RBCs was not enhanced.**

Comparison of the BFU-E colony forming by CD48⁻ CD150⁺ LSK cells isolated from HSCs cultured with or without RBCs. Numbers of BFU-E colonies after 10 days of culture in MethoCult™ (medium with EPO) are shown (*n* = 6 per group). All data are presented as means ± SD. *P*-value is shown in the figure.
